# Steroid Receptor Coactivator 3 Regulates Synaptic Plasticity and Hippocampus-dependent Memory

**DOI:** 10.1007/s12264-021-00741-5

**Published:** 2021-07-06

**Authors:** Hai-Long Zhang, Bing Zhao, Pin Yang, Yin-Quan Du, Wei Han, Jianming Xu, Dong-Min Yin

**Affiliations:** 1 Key Laboratory of Brain Functional Genomics, Ministry of Education and Shanghai, School of Life Science, East China Normal University, Shanghai, 200062 China; 2 MOE Frontiers Center for Brain Science, Institute for Translational Brain Research, Fudan University, Shanghai, 200032 China; 3 Department of Molecular and Cellular Biology, Baylor College of Medicine, Houston, TX 77030 USA

**Keywords:** SRC3, Steroid receptor, Synaptic plasticity, Learning and memory, Hippocampus, N-Methyl-D-aspartate receptor

## Abstract

Steroid hormones play important roles in brain development and function. The signaling of steroid hormones depends on the interaction between steroid receptors and their coactivators. Although the function of steroid receptor coactivators has been extensively studied in other tissues, their functions in the central nervous system are less well investigated. In this study, we addressed the function of steroid receptor coactivator 3 (SRC3) – a member of the p160 SRC protein family that is expressed predominantly in the hippocampus. While hippocampal development was not altered in *Src3*^+/−^ mice, hippocampus-dependent functions such as short-term memory and spatial memory were impaired. We further demonstrated that the deficient learning and memory in *Src3*^+/−^ mice was strongly associated with the impairment of long-term potentiation (LTP) at Schaffer Collateral-CA1 synapses. Mechanistic studies indicated that *Src3*^+/−^ mutation altered the composition of N-methyl-D-aspartate receptor subunits in the postsynaptic densities of hippocampal neurons. Finally, we showed that SRC3 regulated synaptic plasticity and learning mainly dependent on its lysine acetyltransferase activity. Taken together, these results reveal previously unknown functions of SRC3 in the hippocampus and thus may provide insight into how steroid hormones regulate brain function.

## Introduction

Steroid hormones and their receptors play important roles in brain development and function [1], and steroid receptors act in a classic genomic signaling pathway to bind with target DNA and alter gene transcription [2]. In addition, these receptors can act in a rapid, non-genomic manner to activate intracellular signaling pathways [3]. The relative abundance and interactions between steroid receptors and their co-activators determine the cellular responses to steroid hormones [4, 5]. Steroid receptor co-activators (SRCs) have been extensively studied in the fields of cancer, metabolism, endocrinology, and development [6–10], but the functions of SRCs in the adult brain are relatively less well studied [11].

The SRC family of p160 proteins consists of SRC1, SRC2, and SRC3. These coactivators physically interact with steroid receptors, including androgen, progesterone, and estrogen receptors as well as receptors for glucocorticoids, in a ligand-dependent manner [12–14]. SRC1 and SRC2 are widely expressed in several brain regions such as the cortex, hippocampus, amygdala, cerebellum, and hypothalamus [15, 16]; they have been shown to regulate a variety of brain functions such as sexual behaviors [17, 18], energy homeostasis [19, 20], learning and memory [21, 22], and the motor function of the cerebellum [15]. Compared to SRC-1 and SRC-2 whose functions in the brain have been studied [23], the functions of SRC3 in the brain remains largely unknown.

SRC3 (also known as p/CIP, RAC3, AIB1, ACTR, TRAM1, and NCOA3) has been implicated in regulating steroid hormone signaling [24]. Its N-terminus contains a basic helix-loop-helix domain that is required for interaction with transcription factors. The central region of SRC3 contains a nuclear receptor interaction domain (RID) that is responsible for the interaction with nuclear receptors. The C-terminus of SRC3 contains the acetyltransferase (KAT) domain. Unlike SRC1 and SRC2, SRC3 is expressed predominantly in the hippocampus and very sparsely in the hypothalamus [6, 15]. In this study, we addressed the function of SRC3 in synaptic plasticity and hippocampus-dependent memory by virtue of the *Src3*^+/−^ mice.

## Materials and Methods

### Animals

C57BL/6N male mice 7–8 weeks old were used in experiments unless otherwise described. Animals were housed in rooms at 23 °C and 50% humidity under a 12 h light/dark cycle and with food and water available *ad libitum*. All experimental procedures were approved by the Institutional Animal Care and Use Committees of East China Normal University. *Src3*^+/−^ mutant mice were described in a previous study [6] and were backcrossed with C57BL/6N mice for >10 generations before experiments.

### Subcellular Fractions

Subcellular fractions were obtained as described previously [25] with modifications. Mouse brain tissue was homogenized in Buffer A (0.32 mol/L sucrose, 1 mmol/L MgCl_2_, 1 mmol/L PMSF, and a protease inhibitor cocktail). Homogenates were passed through a filter to remove cell debris and centrifuged at 500×g for 5 min in a fixed angle rotor to yield the P1 (nuclear) and S1 (cytosolic) fractions. The P1 fraction was washed in Buffer B containing 10 mmol/L KCl, 1.5 mmol/L MgCl_2_, 10 mmol/L Tris-HCl (pH 7.4) and centrifuged at 500×g for 5 min. The pellets were dissolved in Buffer C containing 20 mmol/L HEPES (pH 7.9), 25% glycerol, 1.5 mmol/L MgCl_2_, 1.4 mol/L KCl, 0.2 mmol/L EDTA, 0.2 mmol/L PMSF (phenylmethanesulfonyl fluoride), 0.5 mmol/L DTT (1, 4 dithiothreitol) and incubated on a shaker at 4 °C for 30 min. After centrifugation at 12,000×g for 10 min, the supernatant of P1 was collected as nuclear proteins. The S1 fraction was centrifuged at 10,000×g for 10 min to yield P2 that contained membranes and synaptosomes, and the cytoplasmic S2. To further purify the postsynaptic fractions, synaptosomes in 0.8 mol/L sucrose were mixed with 1/19 volume of Buffer D containing 200 mmol/L HEPES (pH 7.0), 20% Triton X-100, and 1.5 mol/L KCl. Samples were centrifuged at 20,800×g for 45 min using a fixed angle rotor. The pellets were re-suspended in Buffer E containing 1% Triton X-100 and 75 mmol/L KCl using a Dounce mini-homogenizer and centrifuged again at 20,800×g for 30 min to yield a final pellet—the PSD (postsynaptic density) fraction, which was washed with 20 mmol/L HEPES (pH 7.9) and dissolved in 1 × SDS-PAGE sample buffer.

### Western Blot

Western blotting was performed as described in previous studies [25, 26]. Nuclear and S2 solutions were mixed with 6 × SDS-PAGE sample buffer. P2 and PSD fractions were directly dissolved in 1 × SDS-PAGE sample buffer. Respective subcellular fractions were resolved on SDS-PAGE and transferred to nitrocellulose membranes, which were incubated in Tris-HCl buffer containing 0.1% Tween-20 and 5% milk for 1 h at room temperature before incubation with a primary antibody overnight at 4 °C. After washing, the membranes were incubated with an HRP-conjugated secondary antibody in the same TBS buffer for 1 h at room temperature. Immunoreactive bands were visualized by the ChemiDocTM XRS+ Imaging System (BIO-RAD) using enhanced chemiluminescence (Pierce) and analyzed with ImageJ (NIH). The primary antibodies used were as follows: anti-SRC3 (5765, Cell Signaling, Boston, USA); anti-α-tubulin (3873, Cell Signaling); anti-Synapsin1 (2312, Cell Signaling); anti-Histone H3 (9715, Cell Signaling); anti-Calmodulin (05-173, Millipore, Merck KGaA, Darmstadt, Germany); anti-CaMKIIα (11945, Cell Signaling); anti-phospho-CaMKIIα (pThr^286^) (SAB4300228, Sigma, Merck KGaA); anti-NR2A (04-901, Millipore); anti-NR2B (MAB5778, Millipore); anti-GluR1 (ab109450, Abcam, Cambridge, UK); anti-GluR2 (MABN71, Millipore).

### Immunofluorescence

Brain slices were fixed in 4% PFA, permeabilized with 0.3% Triton-X 100 and 5% BSA in PBS and incubated with primary antibodies at 4 °C overnight. After washing 3 times with PBS, samples were incubated with Alexa Fluor-594-conjugated secondary antibodies (1:500; A-11032, Thermo Fisher, Waltham, USA) for 1 h at room temperature. The samples were mounted with Vectashield mounting medium (Vector) and images were captured using a Leica TCS SP8 confocal microscope or by Leica stimulated emission depletion (STED) microscopy. The following primary antibody were used: rabbit anti-NeuN (Abcam, ab177487); rabbit anti-SRC3 (Cell Signaling, 5765); mouse anti-PSD95 (Millipore, MAB1596); Biocytin (Sigma, B4261); and Alexa Fluor-488-conjugated anti-Biocytin (Thermo Fisher, S32354).

### β-Gal Detection

*Src3*^+/−^ mice were killed and perfused with ice-cold saline. Brain slices embedded in 3% agar were cut into 100 μm slices on a Vibratome (VT 1000S, Leica). β-Gal activity was assessed using an *in situ* β-galactosidase staining kit (Beyotime, RG0039, Shanghai, China) following the manufacturer’s protocol. The slices were washed, mounted in Permount (Thermo Fisher Scientific), and imaged using a microscope with a high-sensitivity camera and equipped with a Plan-Neofluar 5×/0.30 NA objective lens (Leica DMI3000 B).

### Chemical Long-Term Potentiation (LTP)

Chemical LTP stimulation in hippocampal slices was performed as previously described [27]. Briefly, chemical LTP was induced by incubating slices for 15 min in artificial cerebrospinal fluid (aCSF) lacking MgCl_2_ and containing 4 mmol/L CaCl_2_, 100 μmol/L picrotoxin, 50 μmol/L forskolin, and 100 nmol/L rolipram. After stimulation, the slices were homogenized and the total lysates were subjected to Western blotting.

### Electrophysiology

#### Slice Preparation

Male mice (7–8 weeks old) were anesthetized with pentobarbital sodium, decapitated, and the brains were quickly removed and chilled in ice-cold aCSF for an additional 1 min. Transverse hippocampus slices (350 μm) were prepared using a Vibroslice (VT 1000S; Leica, Nussloch, Germany) in ice-cold aCSF. The slices were then incubated in regular aCSF containing (in mmol/L): 126 NaCl, 3 KCl, 1.25 NaH_2_PO_4_, 1.0 MgSO_4_, 2.0 CaCl_2_, 26 NaHCO_3_, and 10 glucose for 30 min at 34 °C for recovery, and then at room temperature (25 ± 1 °C) for an additional 2–8 h. For whole-cell recording, mice were perfused transcardially for 1 min with ice-cold modified solution (in mmol/L): 110 NMDG (N-Methyl-D-glucamine), 2.5 KCl, 10 MgSO_4_, 0.5 CaCl_2_, 1.2 NaH_2_PO_4_, 25 NaHCO_3_, and 25 glucose, to protect neurons and maintain the functional connectivity of brain slices. All solutions were saturated with 95% O_2_/5% CO_2_ (vol/vol).

#### Whole-cell Patch-clamp Recording

CA1 neurons were visualized with infrared optics using an upright microscope equipped with a 40× water-immersion lens (BX51WI; Olympus, Monolith, Japan) and infrared-sensitive CCD camera. The pipettes were filled with the solution (in mmol/L): 105 K-gluconate, 30 KCl, 10 HEPES, 0.3 EGTA, 4 Mg-ATP, 0.3 Na-GTP, and 10 phosphocreatine sodium (pH 7.3, 280 mOsm). For the spontaneous excitatory postsynaptic current (sEPSC) recording, the membrane potential was held at −70 mV under voltage-clamp. The action potential was recorded under current-clamp. Neurons were held at –70 mV, and injected with different currents (duration, 500 ms; increments, ±20 pA from −200 to 580 pA; interval, 10 s). All data were obtained with an EPC10 double patch-clamp amplifier (HEKA Elektronik, Reutlingen, Germany). Data were low-pass filtered at 10 kHz and digitally sampled at 10 kHz with PatchMaster version 2x90.1. Data were analyzed with Neuromatic version 3.0 (http://www.neuromatic.thinkrandom.com, London, England) which runs within Igor pro 6.7.3.2 (WaveMetrics, Poland, USA).

#### LTP Recording and Quantification

Hippocampal slices from 7–8-week-old male mice were placed in a recording chamber and continuously superfused with pre-warmed (32 ± 1 °C) aCSF at 3 mL/min. Field excitatory postsynaptic potentials (fEPSP) were evoked (0.1-ms current pulses at 0.033 Hz) in the CA1 stratum radiatum by stimulating Schaffer collaterals (SCs) with a concentric bipolar stimulating electrode (FHC, Bowdoin, USA) and recorded in current-clamp using an EPC 10 amplifier (HEKA Elektronik) with aCSF-filled glass pipettes (1–5 MΩ). LTP was induced using three trains of theta burst stimulation (10 bursts at 5 Hz, each of 50 pulses at 100 Hz) at inter-train intervals of 30 s [28, 29]. All data were acquired at a 10 kHz sampling rate using Patchmaster version 2x90.1 software (HEKA Elektronik) and filtered off-line at 2 kHz. Analysis was performed with Neuromatic version 3.0 (http://www.neuromatic.thinkrandom.com). Each EPSP trace was normalized to baseline. Two consecutive EPSP traces were averaged to generate a 1-min bin, which generated LTP summary time-course graphs. For LTP of field potentials, the magnitude of LTP was calculated as an average of normalized EPSP amplitudes 50–60 min after TBS.

### Stereotaxic Injection of Adeno-Associated Virus (AAV)

To generate AAVs that express the KAT and RID domains of *SRC3*, we first subcloned the cDNA of the KAT or RID domain from human *SRC3* into the pAAV-Syn1 promoter-EGFP-p2A-MCS-3Flag vector before packaging the AAVs. All the AAVs were generated by OBiO Technology Corp., Ltd.

For virus injection, 5-week-old *Src3*^+/−^ mice were anesthetized with 1% pentobarbital sodium (100 mg/kg, i.p.) and placed in a stereotaxic apparatus (RWD Life Science, Shenzhen, China). Viruses were injected bilaterally into the CA1 regions of hippocampus at the coordinates: anteroposterior −2.7 mm, mediolateral ±2.25 mm, dorsoventral −1.625 mm relative to bregma. Each injection used 0.5 µL AAV and took 5 min. After the injection, the glass pipette was left in place for 5 min to facilitate diffusion of the virus. The injection sites were examined at the end of the experiments, and animals with incorrect injection sites were excluded from the data analysis. Three weeks after AAV injection, mice were subjected to experiments. All surgery was conducted using aseptic techniques.

### Novel Object Recognition

In the habituation phase, individual adult male mice were placed in a chamber (50 cm × 50 cm × 40 cm) and allowed to freely explore the context for 10 min while being recorded by an overhead camera. In the sampling phase, the animal was placed in the same chamber containing two different objects and allowed to explore them for 5 min. To saturate the odor left by the previous mice and to make the mice more relaxed, we placed standard animal bedding in the chamber. Each mouse was tested 1 h after training. In the testing phase, one of the objects was replaced by a new one, and the time spent exploring each of the two objects was separately recorded using ANY-MAZE software. The discrimination index was calculated using the following formula: (time exploring novel object − time exploring familiar object)/(time exploring novel object + time exploring familiar object).

### Morris Water Maze

The Morris water tank consisted of a circular pool (diameter 120 cm, height 50 cm) filled with water maintained at room temperature (25 ± 1 °C) and made opaque with nontoxic white paint. Mice were trained for 5 days with 4 trials per day and 120 s per trial. We used 3 extra-maze visual cues to ensure that visual spatial memory was used by the mouse to find the location of the hidden platform. Twenty-four hours after the last training trial (day 6), the platform was removed and each mouse were given one trial for 60 s of searching (probe test).

### Statistics

All data are shown as the mean ± SEM. Comparisons between two groups were made using the two-sided *t-*test. Comparisons between three or more groups were made using one-way ANOVA analysis followed by Tukey’s *post hoc* test. Data from Sholl analysis, paired-pulse facilitation, I/O curves of action potentials, LTP, locomotion, and the learning curve in Morris water maze were analyzed by two-way ANOVA. Statistically significant difference was indicated as follows: ***P <0.001, **P <0.01, and *P <0.05. The statistical analysis was performed in GraphPad Prism 8.

## Results

### Subcellular Distribution of SRC3 in Mouse Hippocampus

**Fig. 1:**
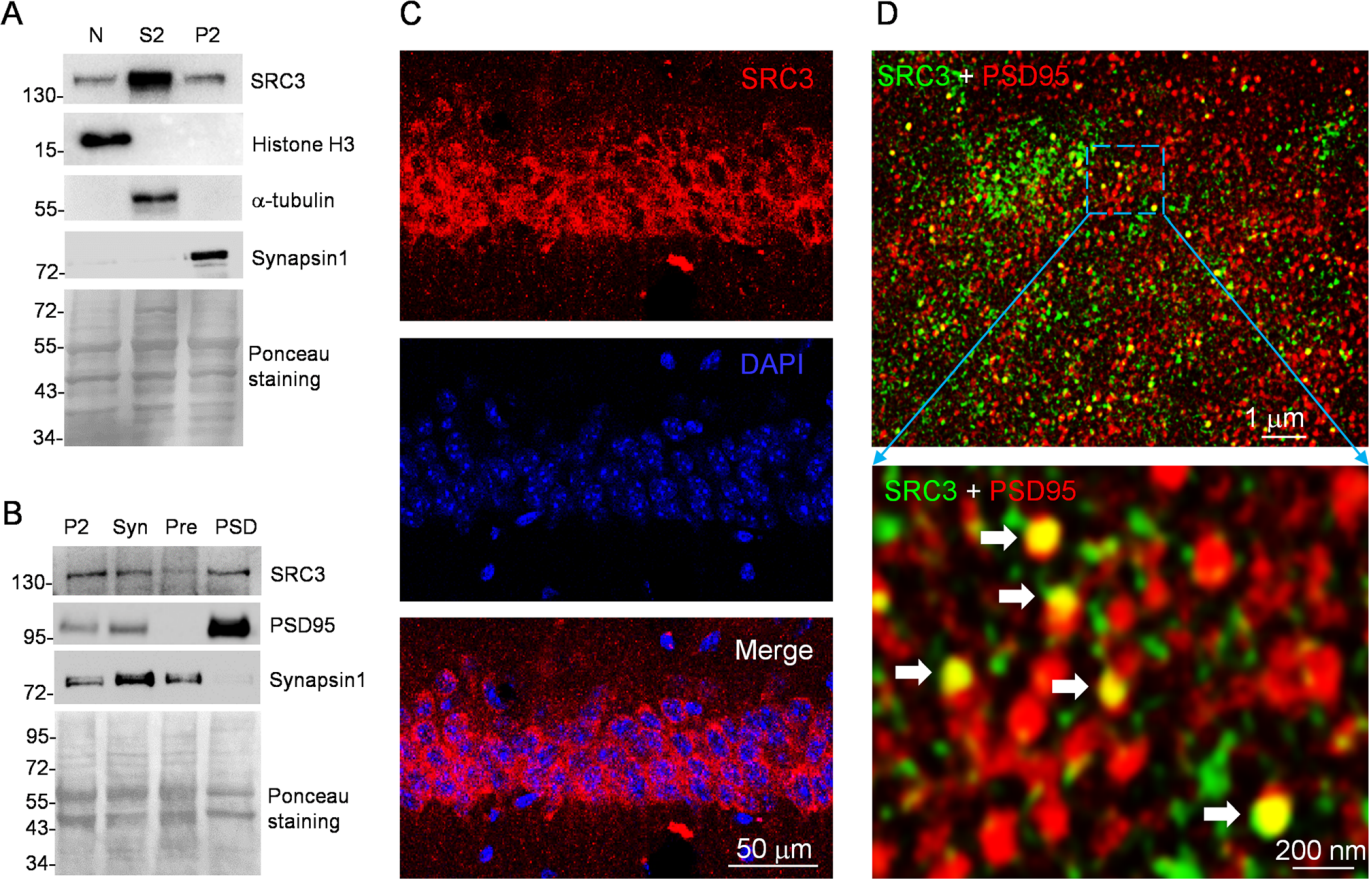
Subcellular distribution of SRC3 in adult mouse hippocampus. **A** Upper panels. expression of SRC3 in the nuclear (N), cytoplasmic (S2) and membrane (P2) fractions. Histone H3, α-tubulin, and synapsin 1 are the protein markers for the N, S2 and P2 fractions. Lower panel, Ponceau staining to show equal protein loading. **B** Upper panels, expression of SRC3 in synaptosome (Syn), presynaptic (Pre), and postsynaptic (PSD) fractions. Synapsin 1 and PSD95 are the protein markers for Pre and PSD fractions. Lower panel, Ponceau staining to show equal protein loading. **C** Confocal microscopic, immunofluorescence images of SRC3 in the mouse CA1 hippocampal region (scale bar, 50 µm). **D** Upper panel, STED microscopic immunofluorescence image of SRC3 and PSD95 in the mouse CA1 hippocampal region (scale bar, 1 µm). Lower panel, enlargement of the rectangle in the upper panel; arrows indicate co-localization of SRC3 and PSD95; scale bar, 200 nm).

To investigate the subcellular distribution of SRC3, we separated adult mouse hippocampal cells into nuclear (N), cytosolic (S2), and membrane (P2) fractions. The purity of these fractions was confirmed by the enrichment of different markers: histone protein H3 in the N, α-tubulin in the S2, and synapsin 1 in the P2 fractions (Fig. 1A). In addition to the nucleus, SRC3 was also expressed in the cytoplasm and P2 fractions (Fig. 1A). To further study the distribution of SRC3 in the synapse, we purified the synaptosomal presynaptic (Pre) and postsynaptic density (PSD) fractions. The purity of the Pre and PSD fractions was evidenced by the enrichment of synapsin 1 in the Pre, and PSD95 in the PSD (Fig. 1B). We found that SRC3 was expressed in the synaptosomes and was more enriched in the PSD than Pre (Fig. 1B).

To examine the distribution of SRC3 in a more direct way, we performed immunofluorescent (IF) assay in the mouse CA1 region. SRC3 was strongly expressed in the cytoplasm in addition to the nucleus (Fig. 1C), consistent with the results of Western blotting (Fig. 1A). To visualize the expression of SRC3 in the Pre and PSD, we performed IF assays using STED microscopy. In agreement with the results from subcellular fractionation, SRC3 was expressed in the PSD (Fig. 1D), suggesting that SRC3 regulates synaptic function.

### Normal Glutamatergic Transmission at the Schaffer Collateral (SC)-CA1 Synapse in *Src3*^+/−^ Mice

**Fig. 2:**
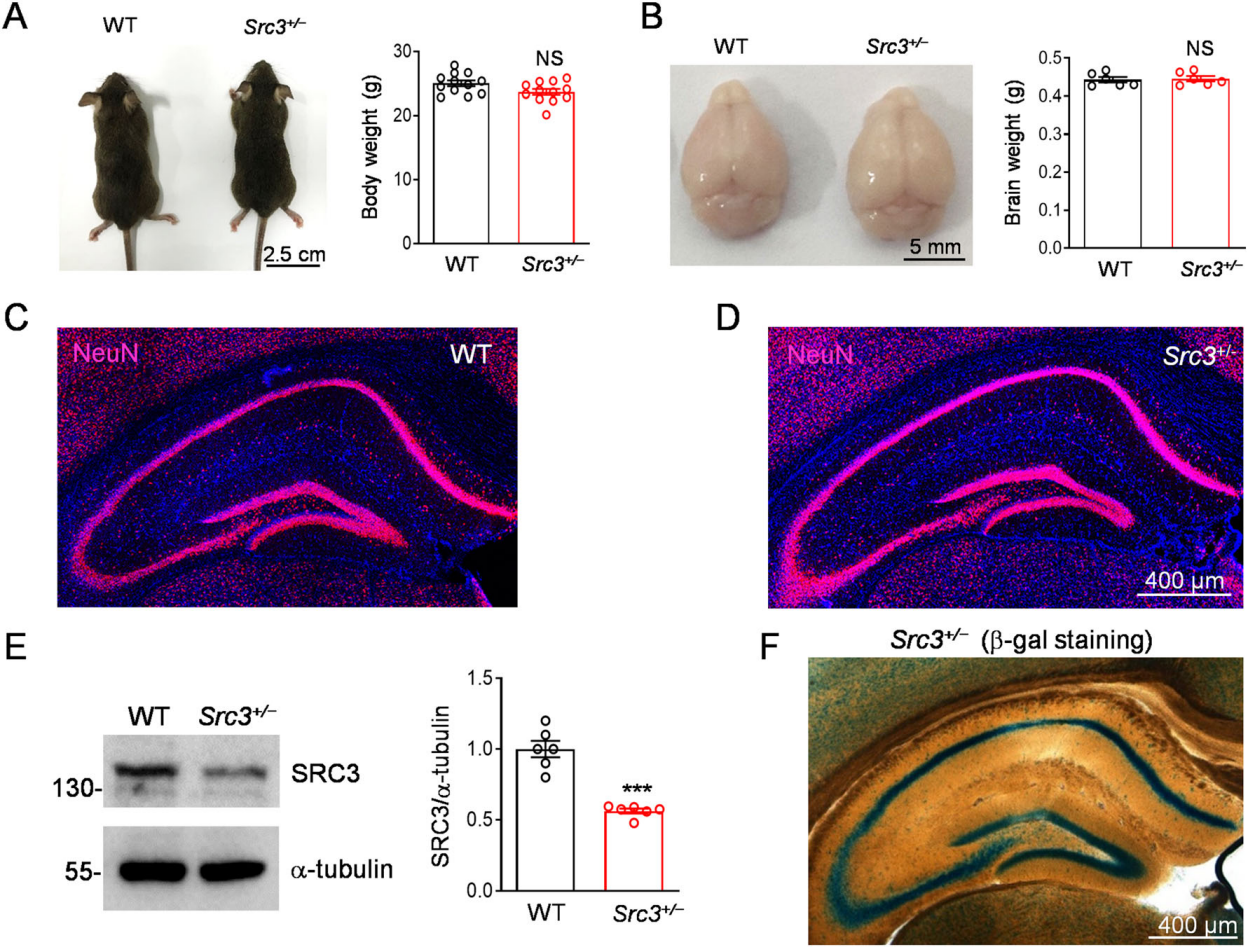
Normal development of *Src3*^*+/−*^ mice. **A** Images (left) and quantification (right) of body weight of 2-month-old *Src3*^+/−^ and WT mice (NS, not significant, two-sided *t-*test; *n =* 12/group; mean ± SEM; scale bar, 2.5 mm0. **B** Images (left) and quantification (right) of brain weight of 2-month-old *Src3*^+/−^ and WT mice (NS, not significant, two-sided *t-*test, *n =* 6/group; mean ± SEM; scale bar, 5 mm). **C, D** Hippocampal slices from 2-month-old WT (**C**) and *Src3*^*+/−*^ mice (**D**) stained with anti-NeuN (pink) and DAPI (blue) showing intact global morphology in *Src3*^+/−^ mice (scale bar, 400 µm). **E**, Representative blots (left) and quantification (right) of SRC3 protein/α-tubulin levels in hippocampi from *Src3*^+/−^ and WT mice (*n =* 6/group; mean ± SEM normalized to WT; ***P <0.001, two-sided *t-*test). **F** Representative image of β-gal activity in a 2-month-old *Src3*^+/−^ hippocampus (scale bar, 400 µm).

To investigate the function of SRC3 in the synapse, we characterized *Src3* mutant mice in which a cassette containing lacZ is inserted into the *Src3* gene [6]. Because the *Src3*-null mutation causes overall growth retardation and partial lethality [6], we focused on *Src3*^+/−^ mice that were normal in body and brain weights and did not display global morphological deficits in the hippocampus (Fig. 2A–D). However, the SRC3 protein levels were reduced to about half in the hippocampus of *Src3*^+/−^ mice compared with wild-type (WT) littermates (Fig. 2E). β-Gal, which is expressed under the promoter of the *Src3* gene, was high in the stratum pyramidale layer of the hippocampus where the somata of pyramidal neurons are enriched (Fig. 2F), in agreement with a previous report [6].

**Fig. 3:**
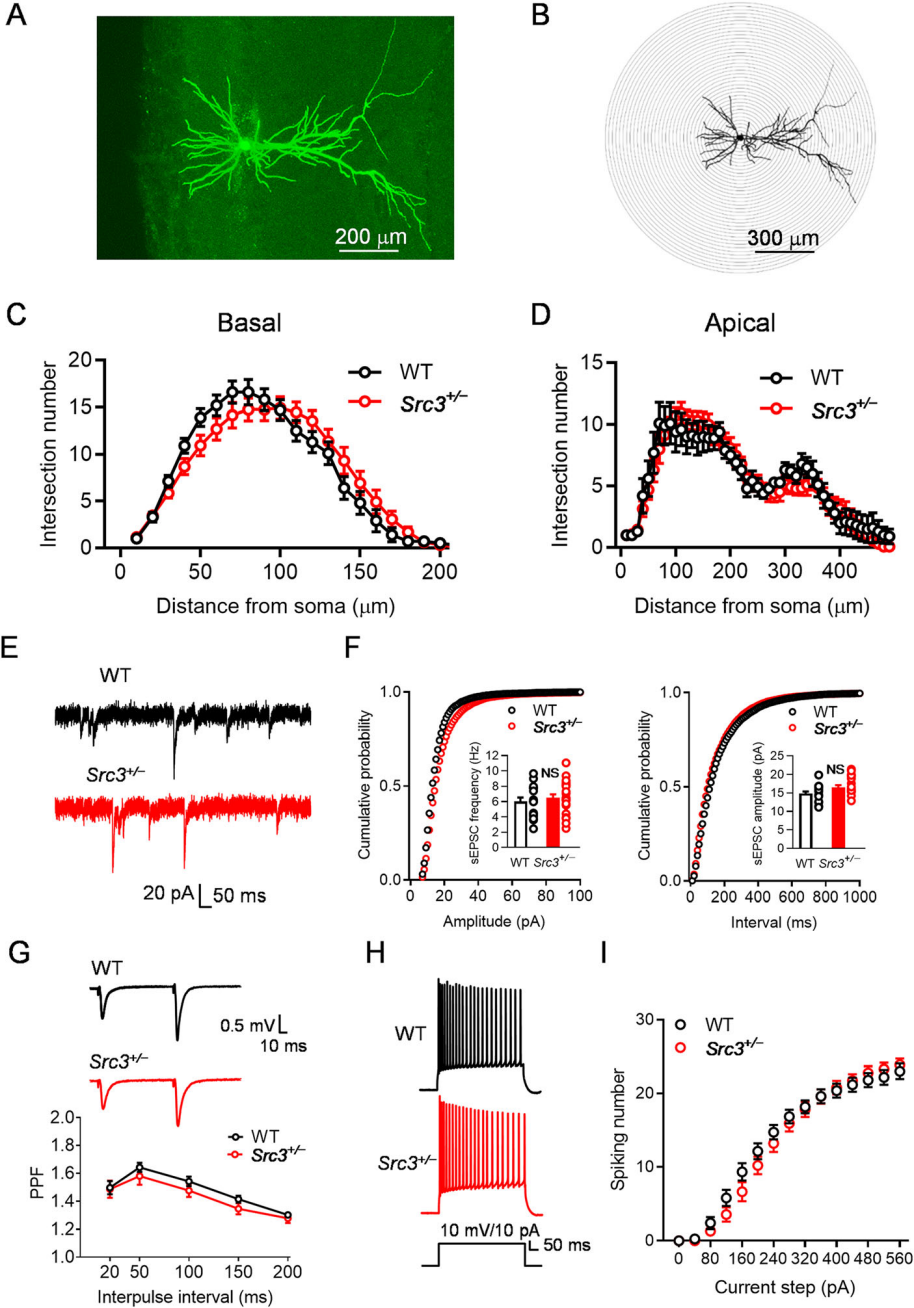
Intact basal glutamatergic transmission in CA1 pyramidal neurons from *Src3*^*+/−*^ hippocampus. **A** Dendritic morphology of a CA1 pyramidal neuron from a 2-month-old WT mouse; the neuron was filled with biocytin during recording and later fixed and immunostained with anti-biocytin antibodies (scale bar, 200 µm). **B** Diagram for Sholl analysis of the neuron in **A** (scale bar, 300 µm). **C** Basal dendritic arborization of CA1 pyramidal neurons is not altered in *Src3*^+/−^ mice (*n =* 10 cells from 4 WT mice, *n =* 13 cells from 4 *Src3*^+/−^ mice; mean ± SEM; Genotype *F*_(1, 21)_ = 0.0000118, *P =* 0.9973, two-way ANOVA). **D** Apical dendritic arborization of CA1 pyramidal neurons is not altered in *Src3*^+/−^ mice (*n =* 10 cells from 4 WT mice, *n =* 13 cells from 4 *Src3*^+/−^ mice, mean ± SEM; Genotype *F*_(1, 21)_ = 0.1046, *P =* 0.7496, two-way ANOVA). **E** Representative sEPSCs in CA1 pyramidal neurons from WT and *Src3*^+/−^ mice. **F** Similar cumulative plots of sEPSC frequency (left) and amplitude (right) in CA1 pyramidal neurons from WT and *Src3*^+/−^ mice (quantification in inserts; *n =* 16 cells from 4 WT mice, *n =* 18 cells from 4 *Src3*^+/−^ mice; mean ± SEM; NS, not significant, two-sided *t-*test. **G** Representative traces (upper) and quantification (lower) showing similar paired-pulse facilitation (PPF) at SC-CA1 synapses in WT and *Src3*^+/−^ mice (*n =* 12 slices from 4 WT mice, *n =* 9 slices from 4 *Src3*^+/−^ mice; mean ± SEM; Genotype *F*_(1, 95)_ = 3.271, *P =* 0.0737, two-way ANOVA). **H** Representative action potentials in CA1 pyramidal neurons from WT (upper) and *Src3*^+/−^ (lower) mice. **I** I/O curves of CA1 pyramidal neurons showing comparable excitability of CA1 pyramidal neurons in WT and *Src3*^+/−^ mice (*n =* 15 cells from 4 WT mice, *n =* 19 cells from 4 *Src3*^+/−^ mice; mean ± SEM; Genotype *F*_(1, 32)_ = 0.1999, *P =* 0.6578, two-way ANOVA.

We first studied whether *Src3*^+/−^ mutation affects dendritic arborization in hippocampal CA1 pyramidal neurons. To this end, we filled the neurons with biocytin during electrophysiological recording and used immunofluorescence to reveal the dendritic morphology (Fig. 3A). The extent of dendrite arborization was quantified by Sholl analysis (Fig. 3B). The arborization of basal and apical dendrites of CA1 pyramidal neurons was similar in the WT and *Src3*^+/−^ mice (Fig. 3C, D), which suggest that *Src3*^+/−^ mutation does not affect dendritic development.

To study whether *Src3*^+/−^ mutation alters glutamatergic transmission, we used whole-cell patch clamp to record spontaneous EPSCs (sEPSCs) in CA1 pyramidal neurons. As shown in Fig. 3E and F, both the frequency and amplitude of sEPSCs were comparable in the WT and *Src3*^+/−^ mice, indicating that glutamatergic transmission is normal in *Src3*^+/−^ mice. Consistent with this result, the probability of glutamate release indicated by paired-pulse facilitation at SC-CA1 synapses was not altered in *Src3*^+/−^ mice (Fig. 3G). Likewise, the excitability of CA1 pyramidal neurons was also normal in *Src3*^+/−^ mice (Fig. 3H, I). Taken together, these results indicate that glutamatergic transmission is not altered by *Src3*^+/−^ mutation.

### An Important Role of SRC3 in Synaptic Plasticity

**Fig. 4:**
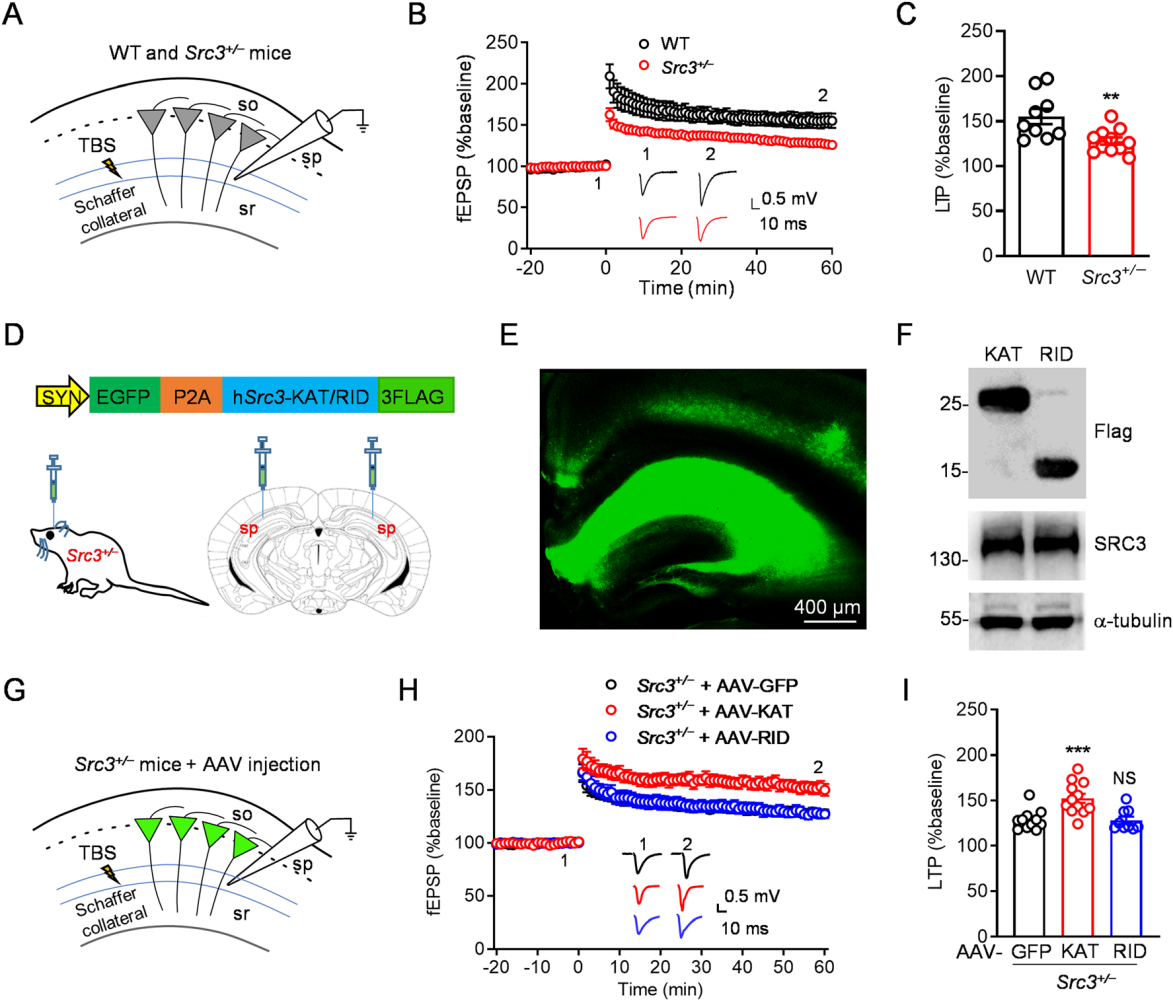
Importance of SRC3 in LTP at the SC-CA1 synapse. **A** Diagram of fEPSP recording at SC-CA1 synapses from WT and *Src3*^+/−^ mice. **B** Normalized fEPSP amplitude *versus* time (1-min intervals) for WT and *Src3*^+/−^ hippocampal slices. **C** Quantification of data as in **B** showing reduced LTP in *Src3*^+/−^ slices compared with control slices (*n =* 9 slices from 5 WT mice, *n =* 11 slices from 5 *Src3*^+/−^ mice; mean ± SEM; **P = 0.0066, two-sided *t-*test). **D** Upper, schematic of AAV construct expressing EGFP-P2A-h*Src3* KAT/RID-3FLAG (SYN, human synapsin 1 promoter. Lower, diagram of stereotaxic injection of AAV into the hippocampus of the *Src3*^+/−^ mouse. **E** Expression of EGFP in the hippocampus 3 weeks after AAV injection (scale bar, 400 µm). **F** Expression of Flag, SRC3, and α-tubulin in the hippocampus 3 weeks after AAV injection. **G** Diagram of fEPSP recording at SC-CA1 synapses in the *Src3*^+/−^ hippocampus infected by AAV as in panel **D**. **H** Normalized fEPSP amplitude *versus* time (1-min intervals) in hippocampal slices infected by the indicated AAV. **I** Quantification of data as in **H** showing that the KAT domain but not the RID of SRC3 rescues the LTP deficit in *Src3*^+/−^ mice (*n =* 11 slices from 5 *Src3*^+/−^ mice overexpressing GFP, *n =* 12 slices from 6 *Src3*^+/−^ mice overexpressing SRC3-KAT, *n =* 9 slices from 5 *Src3*^+/−^ mice overexpressing SRC3-RID; mean ± SEM; NS, not significant, ***P = 0.0007, one-way ANOVA *vs* GFP group).

Next, we investigated whether *Src3*^+/−^ mutation affects synaptic plasticity. The N-methyl-D-aspartate receptor (NMDAR)-dependent LTP at the SC-CA1 synapse is one of the most common forms of synaptic plasticity [30–32]. We recorded theta burst stimulation (TBS)-induced LTP at the SC-CA1 synapse from WT and *Src3*^+/−^ hippocampal slices (Fig. 4A). Remarkably, TBS-elicited LTP at SC-CA1 synapses was impaired in *Src3*^+/−^ mice compared with WT littermates (Fig. 4B, C), indicating an important role of SRC3 for LTP. SRC3 has a RID domain that interacts with the steroid hormone receptor and a KAT domain that exhibits lysine acetyltransferase activity [24]. To further determine whether the RID or KAT domain of SRC3 can rescue the LTP deficit in *Src3*^+/−^ mice, we generated AAVs that expressed GFP or GFP plus a Flag-tagged RID or KAT domain of SRC3 (Fig. 4D). We delivered the AAV by stereotaxic injection into the hippocampus of 5-week-old *Src3*^+/−^ mice, and 3 weeks later the EGFP and Flag-tagged proteins were detected in the hippocampus (Fig. 4E, F). The TBS-induced LTP at the SC-CA1 synapse was recorded from *Src3*^+/−^ mice after hippocampal injection of AAV (Fig. 4G). Intriguingly, the LTP deficit in *Src3*^+/−^ hippocampal slices was rescued by the KAT domain, but not the RID of SRC3 (Fig. 4H, I), suggesting that the KAT domain but not the RID of SRC3 is important for LTP.

### Alteration of NMDAR Subunits in the Hippocampus of *Src3*^+/−^ Mice

**Fig. 5:**
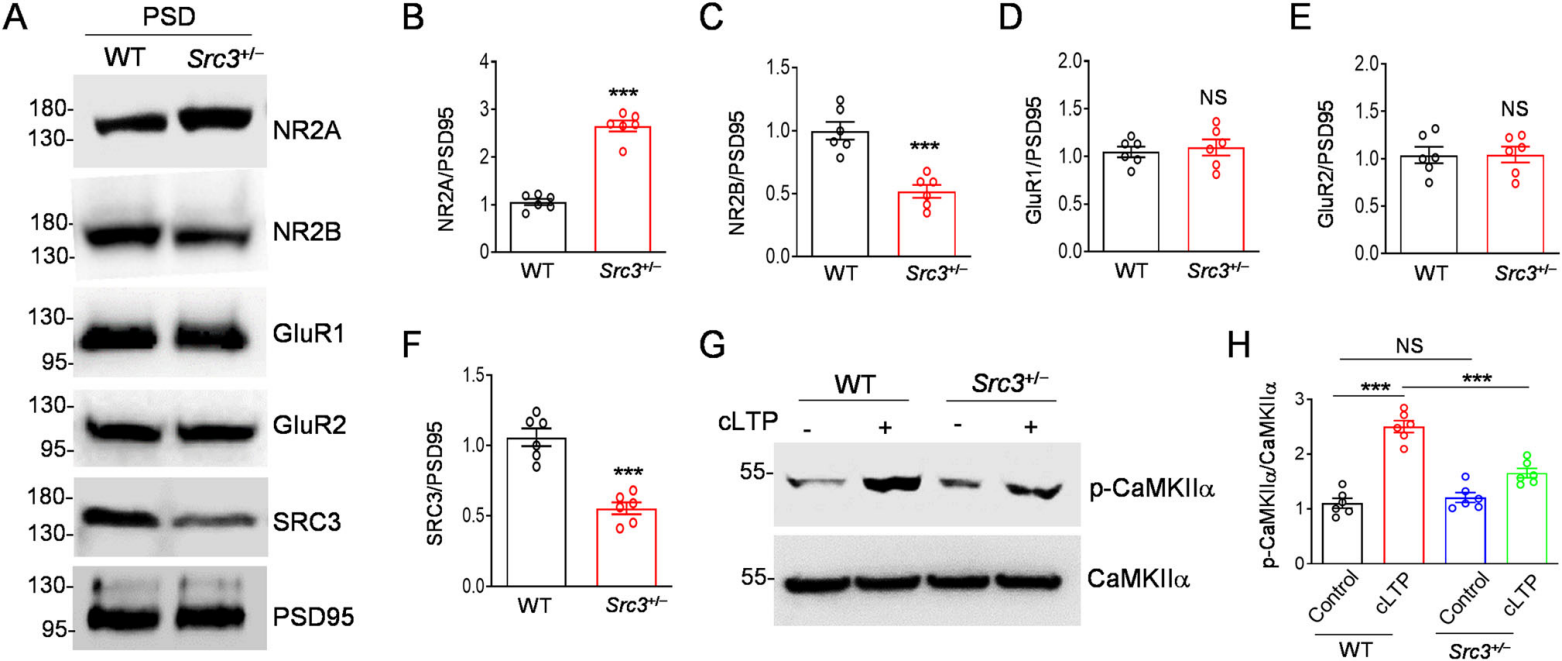
Alteration of NMDAR subunits in the PSD of the *Src3*^+/−^ hippocampus. **A** Western blots showing increased NR2A subunits but decreased NR2B subunits of NMDARs in the PSD of the *Src3*^+/−^ hippocampus, compared with the WT hippocampus. **B** Quantification of NR2A/PSD95 as in panel **A** (*n =* 6/group; mean ± SEM; ***P <0.001, two-sided *t-*test). **C** Quantification of NR2B/PSD95 as in panel **A** (*n =* 6/group; mean ± SEM; ***P = 0.0003). **D** Quantification of GluR1/PSD95 as in panel **A** (*n =* 6/group; mean ± SEM; NS, not significant, two-sided *t-*test. **E** Quantification of GluR2/PSD95 as in panel **A** (*n =* 6/group; mean ± SEM; NS, not significant, two-sided *t-*test). **F** Quantification of SRC3 protein as in panel **A** s normalized to the WT showing reduced levels in the hippocampus from *Src3*^+/−^ mice (*n =* 6/group; mean ± SEM; ***P <0.001, two-sided *t-*test). **G** Western blots showing reduced cLTP-induced CaMKIIα activation in *Src3*^+/−^ slices, compared with WT slices. **H** Quantification of p-CaMKIIα/CaMKIIα normalized to WT slices under control conditions as in panel **G** (*n =* 6; mean ± SEM; NS, not significant, ***P <0.001, one-way ANOVA.

Next, we investigated the molecular mechanisms underlying the LTP deficit in *Src3*^+/−^ mice. The NMDAR-dependent AMPAR (α-amino-3-hydroxy-5-methyl-4-isoxazolepropionic acid receptor) trafficking is considered the most important mechanism for LTP [33]. For this reason, we investigated whether the synaptic distribution of NMDARs or AMPARs is altered in *Src3*^+/−^ mice. We purified the PSD fraction from WT and *Src3*^+/−^ hippocampi and performed Western blots on their lysates. As shown in Fig. 5A and B, the NR2A subunit of the NMDAR was higher in the PSD fraction from the *Src3*^+/−^ hippocampus than in controls. In contrast, the NR2B subunit of the NMDAR was lower in the PSD fraction from the *Src3*^+/−^ hippocampus than in WT littermates (Fig. 5A, C). We did not find any difference in the PSD distribution of the AMPAR subunits GluR1 and GluR2 between the WT and *Src3*^+/−^ hippocampus (Fig. 5A, D, E). The SRC3 protein levels were reduced by half in the hippocampus of *Src3*^+/−^ mice compared with WT littermates (Fig. 5A, F). Given that the NR2B subunit of the NMDAR is required for LTP while the NR2A subunit inhibits LTP [34], our results suggest that the alteration of NMDAR subunits in the PSD fraction are responsible for the LTP deficits in the *Src3*^+/−^ hippocampus. In support of this hypothesis was the finding that activation of CaMKIIα, a protein kinase critical for LTP and downstream of NMDARs, was impaired after chemical LTP stimulation in *Src3*^+/−^ hippocampal slices compared with control slices (Fig. 5G, H). Altogether, these data suggest that abnormal composition of NMDAR subunits in the PSD region accounts for the LTP deficit in the hippocampus of *Src3*^+/−^ mice.

### Importance of SRC3 in Hippocampus-dependent Learning and Memory

**Fig. 6:**
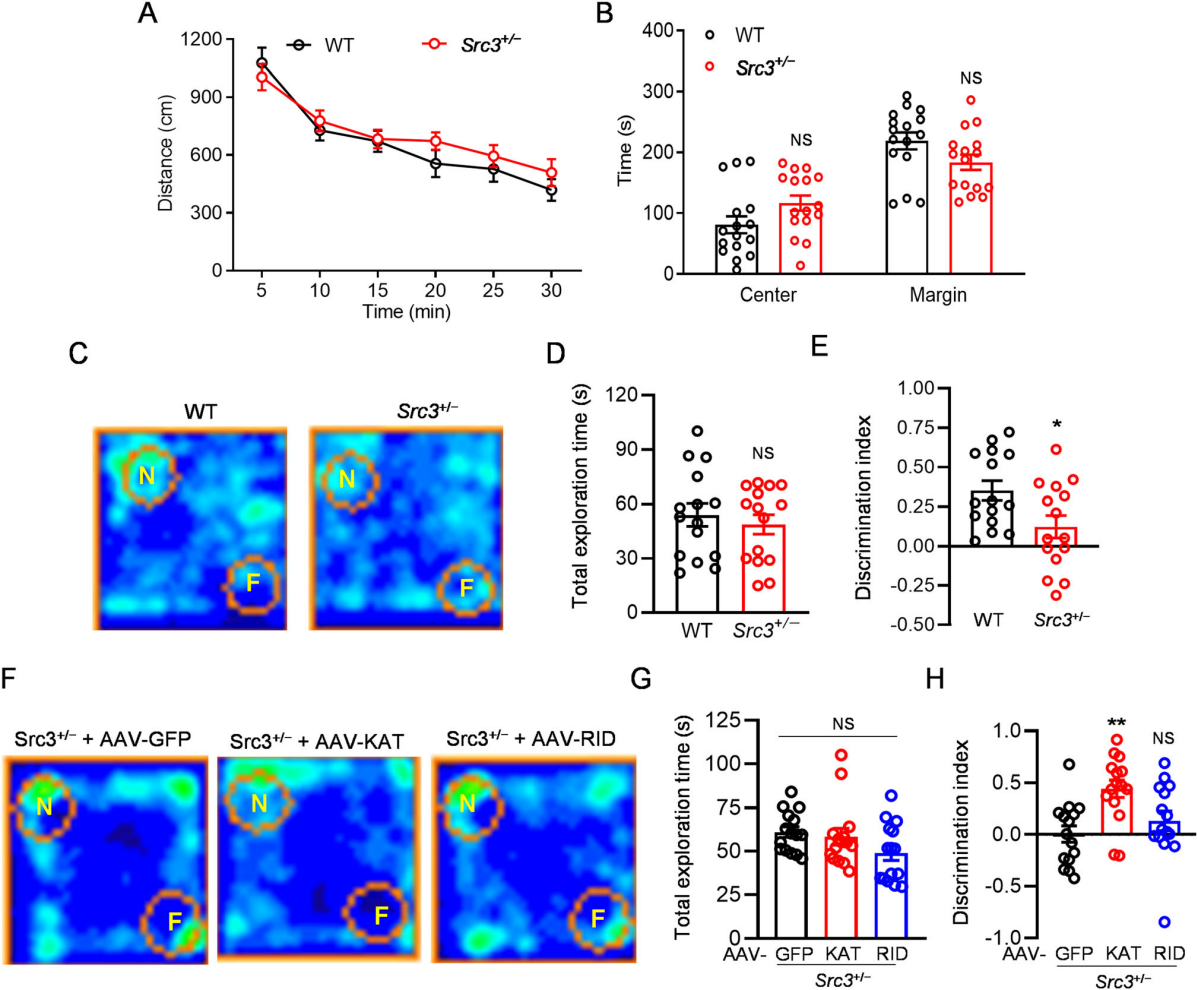
A critical role of SRC3 in novel object recognition (NOR). **A** Distance travelled during the first 30 min in the open field by WT and *Src3*^+/−^ mice (*n =* 16/group; mean ± SEM; Genotype *F*_(1, 30)_ = 0.4119, *P =* 0.5259). **B** Time spent in the center and margin of the open field during the first 5 min by WT and *Src3*^+/−^ mice (*n =* 16/group; mean ± SEM; NS, not significant, two-sided *t-*test. **C** Occupancy plot of the heads of WT and *Src3*^+/−^ mice around a novel (N) and a familiar (F) object. **D** Total exploration time with N and F in WT and *Src3*^+/−^ mice (*n =* 15/group; mean ± SEM; NS, not significant, two-sided *t-*test. **E** Discrimination indexes showing impaired NOR in *Src3*^+/−^ mice (*n =* 15/group; mean ± SEM; *P = 0.0218, two-sided *t-*test. **F** Occupancy plot of the heads of *Src3*^+/−^ mice receiving different AAV treatment around a novel (N) and a familiar (F) object. **G** Total exploration time with N and F in *Src3*^+/−^ mice receiving different AAV treatments (*n =* 15/group; mean ± SEM; NS, not significant, one-way ANOVA. **H** Discrimination index showing the KAT domain but not the RID of SRC3 rescues the NOR deficit of *Src3*^+/−^ mice (*n =* 15/group; mean ± SEM; NS, not significant, **P = 0.0017, one-way ANOVA *vs* GFP group).

LTP at SC-CA1 synapses is coupled to hippocampus-dependent learning and memory [21, 35–38], thus we next investigated whether SRC3 is important for these processes. No difference was found between WT and *Src3*^+/−^ mice in locomotor activity in the open field (Fig. 6A), indicating no deficits in the locomotion of these mice. The time spent in the center and margin of the open field was also similar in WT and *Src3*^+/−^ mice (Fig. 6B), suggesting no anxiety-like behavior in these mice.

Next, we studied whether SRC3 is important for novel object recognition (NOR), a paradigm for investigating hippocampus-dependent short-term memory [39]. Although the total exploration time around novel and familiar subjects was similar in the WT and *Src3*^+/−^ mice (Fig. 6C, D), the discrimination index was significantly lower in the *Src3*^+/−^ mice than in WT littermates (Fig. 6C, E). These results suggest that SRC3 is important for NOR. To address whether the KAT or RID domain of SRC3 is critical for NOR, we delivered AAVs expressing GFP, KAT, or RID of SRC3 by stereotaxic injection into the hippocampus of 5-week-old *Src3*^+/−^ mice. Strikingly, 3 weeks later, the NOR deficit in *Src3*^+/−^ mice was rescued by the AAV expressing KAT but not that expressing RID (Fig. 6F–H), similar to the rescue of LTP (Fig. 4G–I). These data indicate that SRC3 regulates NOR through the KAT domain.

**Fig. 7:**
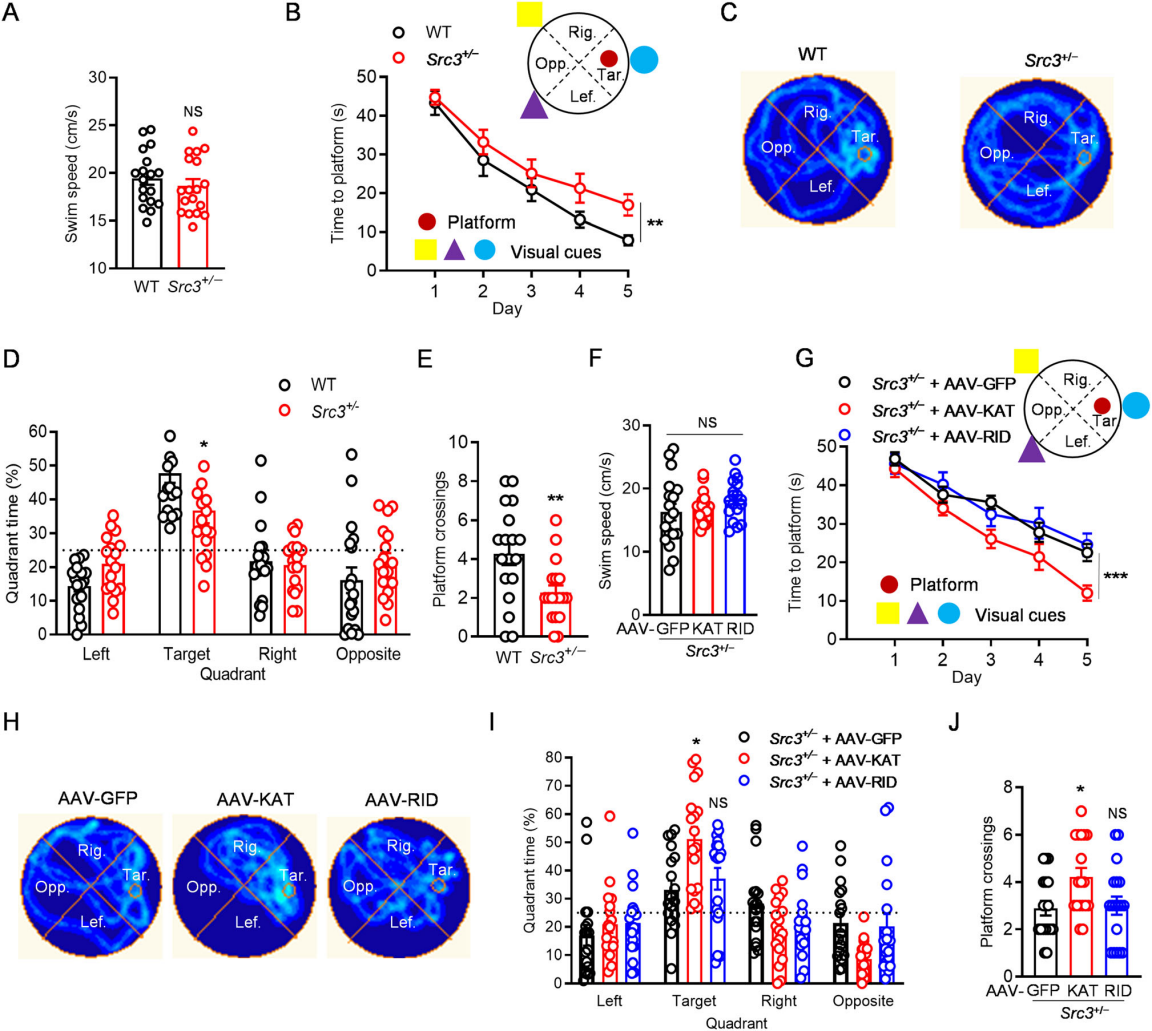
Importance of SRC3 in spatial memory in the Morris water maze (MWM). **A** Swimming speeds in WT and *Src3*^+/−^ mice (*n =* 18/group; mean ± SEM; NS, not significant, two-sided *t-*test). **B** Learning curves in *Src3*^+/−^ and WT mice during training (*n =* 18/group; mean ± SEM; **Genotype *F*_(1, 170)_ = 8.842, *P =* 0.0041, two-way ANOVA, Tar, target quadrant; Opp, opposite quadrant; Lef, left quadrant; Rig, right quadrant). **C** Swimming traces of WT and *Src3*^+/−^ mice during probe tests. **D** Times spent in the target quadrant by *Src3*^+/−^ and WT mice (*n =* 18/group; mean ± SEM; *P = 0.0216, two-way ANOVA followed by Sidak’s multiple comparison test). **E** Platform crossings in the *Src3*^+/−^ and WT mice during probe tests (*n =* 18/group; mean ± SEM; **P = 0.0064, two-sided *t-*test). **F** Swimming speeds in *Src3*^+/−^ mice receiving indicated AAV treatment (*n =* 18/group; mean ± SEM; NS, not significant, one-way ANOVA). **G** Time to platform showing the KAT domain but not the RID of SRC3 rescues the learning deficit of *Src3*^+/−^ mice (*n =* 15/group; mean ± SEM; ***Genotype *F*_(2, 225)_ = 11.23, *P <*0.001, two-way ANOVA). **H** Swimming traces of *Src3*^+/−^ mice receiving different AAV treatment during probe tests. **I** Time spent in the target quadrant during probe tests showing the KAT domain but not the RID of SRC3 rescues the memory deficit of *Src3*^+/−^ mice (*n =* 18/group; mean ± SEM; *P = 0.0283, two-way ANOVA followed by Tukey’s multiple comparison test). **J** Numbers of platform-crossings during probe tests showing that the KAT domain but not the RID of SRC3 rescues the memory deficit of *Src3*^+/−^ mice (*n =* 18/group; mean ± SEM; NS, not significant, *P = 0.0218, one-way ANOVA *vs* GFP group).

Lastly, we investigated whether SRC3 is important for hippocampus-dependent spatial memory in the Morris water maze [40, 41]. Although the swimming speed was similar in WT and *Src3*^+/−^ mice (Fig. 7A), the learning process was slower in *Src3*^+/−^ mice than in WT littermates (Fig. 7B). During probe tests, the time spent in the target area and the number of platform crossings were significantly lower in *Src3*^+/−^ mice than in WT littermates (Fig. 7C–E). These results indicate that SRC3 is important for hippocampus-dependent spatial memory. Remarkably, the water-maze deficits in *Src3*^+/−^ mice were rescued by the hippocampal injection of AAVs expressing the KAT domain but not RID of SRC3 (Fig. 7F–J), similar to the rescue of NOR (Fig. 6F–H). Taken together, these results demonstrate that SRC3 is important for hippocampus-dependent learning and memory.

## Discussion

In this study, we demonstrated a previously unknown function of SRC3 in regulating synaptic plasticity and hippocampus-dependent learning and memory. SRC3 is known to be expressed in the brain; however, its function in the brain is largely unknown. We showed here that *Src3* mRNA is enriched in the pyramidal neurons of the hippocampus, in agreement with a previous report [6] and data from the Allen Brain Institute (https://mouse.brain-map.org/). An early study reported that female but not male *Src3* mutant mice show increased anxiety in the open field test [42], consistent with our finding. Although a previous study indicated that knockdown of SRC3 by shRNA increases dendritic arborization in cultured hippocampal neurons [43], our results suggested that dendritic arborization is not altered in the CA1 pyramidal neurons of *Src3*^*+/−*^ mice. The discrepancy between these two studies might be a result of the different approaches to down-regulating SRC3 (shRNA *versus* heterozygous mutation), or the different conditions *in vitro* and *in vivo*.

SRC3 is critically involved in the classical genomic signaling of steroid hormones [24]. On the other hand, steroid hormones can act in a non-classical manner through receptors on the cell membrane [44]. We showed here that SRC3 is strongly expressed in the cytoplasm and synapses, in addition to the nucleus in the mouse hippocampus. These findings suggest that SRC3 may regulate both the genomic and non-genomic signaling pathways of steroid hormones. The synaptic distribution of NMDAR subunits and the downstream kinase activation were impaired in *Src3*^+/−^ mice, suggesting crosstalk between steroid hormone signaling and NMDAR expression or function. In support of this hypothesis are previous studies showing that steroid hormones such as estrogen and progesterone regulate the expression or function of NMDARs in the brain [45–47].

*Src3*^*−/−*^ mice exhibit growth retardation while *Src3*^+/−^ mice show the same growth rate as WT littermates [6]. The exact reasons why *Src3*^+/−^ mice have a normal body weight and length are not clear. The SRC family of proteins (SRC1, SRC2, and SRC3) are all expressed in the nucleus to regulate gene expression. We speculate that half of SRC3 protein level might be sufficient for the regulation of gene expression required for body growth, along with compensation by SRC1 or SRC2.

We also demonstrated that the KAT domain rather than the RID of SRC3 is critical for regulating synaptic plasticity and memory. The RID of SRC3 contains three LXXLL (X represents any amino-acid) motifs, which form amphipathic α-helices and are responsible for binding with nuclear receptors [24]. The KAT domain is localized in the C terminus of SRC3 and exhibits histone acetyltransferase activity [48], although its cellular substrates are incompletely identified.

Our results support the importance of the lysine acetyltransferase activity of SRC3 in regulating brain functions. Apart from histone proteins in the nucleus, the substrates of SRC3 in the brain remain largely unknown. Given that SRC3 is expressed in the cytoplasm and synapses, it is possible that SRC3 may acetylate cytoplasmic or synaptic proteins. Recent studies indicate that *Src3* is genetically linked with some neurodegenerative disorders such as multiple sclerosis and hearing loss [49, 50]. Future work is required to address how dysregulation of *Src3* leads to brain disorders.
